# Supplementary material for: Dual Targeting of CDK4/6 and cMET in Metastatic Uveal Melanoma
Source: Cancers (Basel). 2021 Mar 4;13(5):1104. doi: 10.3390/cancers13051104 (PMC7961994; doi:10.3390/cancers13051104)
Supplement: Supplementary file 1 [file cancers-13-01104-s001.pdf]

cancers

MDPI

Supplementary materials

Dual targeting of CDK4/6 and cMET in metastatic uveal melanoma

Masahiro Ohara *, Kengo Saito, Ken Kageyama, Mizue Terai, Hanyin Cheng, Andrew E. Aplin and Takami Sato

1. Quantitative real-time polymerase chain reaction

RNA was extracted from samples with RNeasy Mini Kit (Qiagen, Germantown, Maryland, USA) and melanin removed from extracted RNA with the One Step PCR Inhibitor Removal Kit (Zymo Research, Irvine, California, USA) according to manufacturer's instructions. Complementary DNA was prepared by reverse transcription PCR using the SuperScript VILO MasterMix kit (Thermo Fisher Scintific, Waltham, Massachusetts, USA). Quantitative real-time reverse transcription-polymerase chain reaction (qPCR) analysis of expression of the *TOPO2* and *TK1* genes was performed using PowerUp SYBR Green Master Mix (Thermo Fisher Scintific) with an ABI Prism 7900HT sequence detection system. The sequences of the qPCR primers were as follows: *TOPO2*, 5'-aatctcagagcttcccgtca-3' (forward) and 5'-tgcctctgccagtttttctt-3' (reverse); *TK1*, 5'-ggcag-tttttccctgacatc-3' (forward) and 5'-cctcgacctccttctctgtg-3' (reverse); and *glyceraldehyde-3-phosphate dehydrogenase* (*GAPDH*), 5'-gagtcaacggatttggtcgt-3' (forward) and 5'-ttgatttt-ggagggatctcg-3' (reverse).

2. Mutation analysis

DNA was extracted from the samples with the QIAmp DNA Mini Kit (Qiagen), and melanin was removed from extracted DNA with the One Step PCR Inhibitor Removal Kit (Zymo Research) according to manufacturer's instructions. The mutational status of the *GNAQ*, *GNA11*, *BAP1*, *SF3B1*, and *E1F1AX* genes were assessed with the custom targeted sequencing panel (Illumina, San Diego, California, USA) using the Miseq sequencer (Illumina). Primer sequences and locations are available on request.

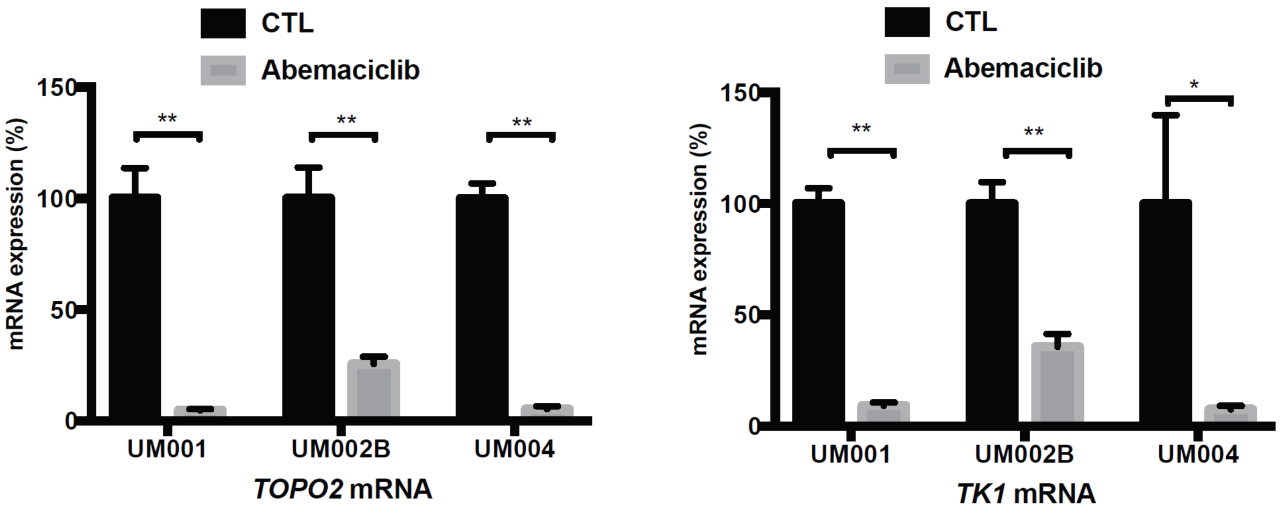

Figure S1. Gene expression downstream of the RB/E2F pathway. RT-qPCR data of UM001, UM002B, and UM004 cells showing the expression levels of E2F target genes, *TOPO2* and *TK1*, after 48 h of treatment with DMSO or 1 µM abemaciclib. Error bars represent the standard error of the mean ($n = 3$). *, $p < 0.05$; **, $p < 0.01$, based on the two sample *t*-test with unequal variance.

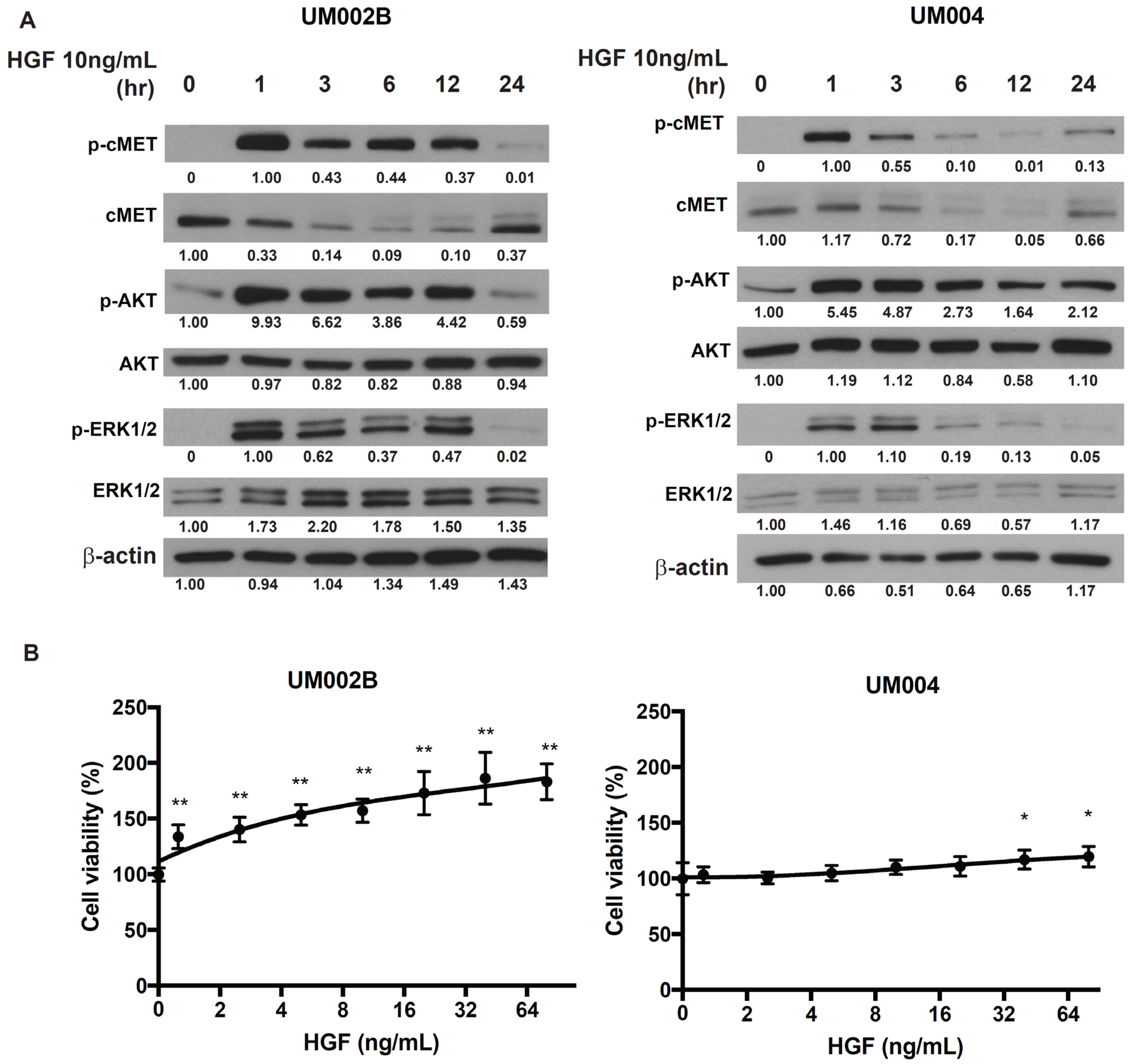

Figure S2. The effect of HGF in two metastatic uveal melanoma cell lines. (**A**) UM002B and UM004 cells were treated with 10 ng/ml of HGF for indicated times. Cells were lysed and Western immunoblotted with the indicated antibodies for phosphorylation of cMET, AKT, and ERK1/2. (B) UM002B and UM004 cells were treated with different concentrations of HGF as indicated for 96 h. Cell viability was analyzed by the MTS assay. Data represent mean cell viability ± SD. *$p < 0.05$; **$p < 0.01$; compared with the group treated with control vehicle based on the two sample *t*-test with unequal variance.

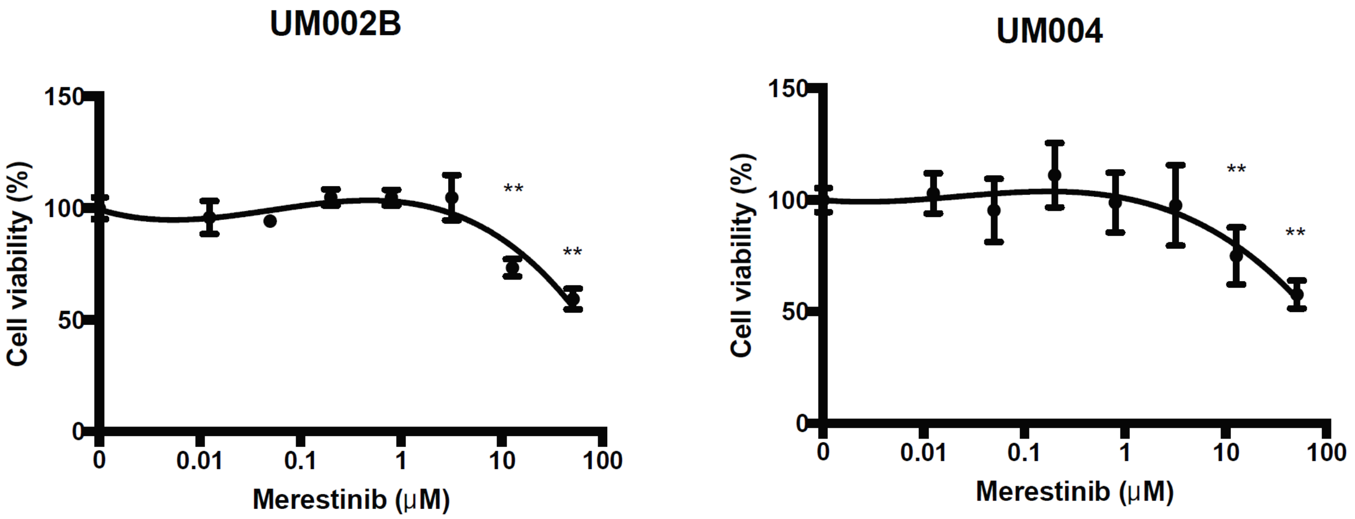

Figure S3. The effect of merestinib in two metastatic uveal melanoma cell lines. UM002B and UM004 cells were treated with various concentrations of HGF as indicated for 96 h. Data represent mean cell viability ± SD. *P < 0.05; **P < 0.01; compared with the group treated with DMSO based on the two sample *t*-test with unequal variance.

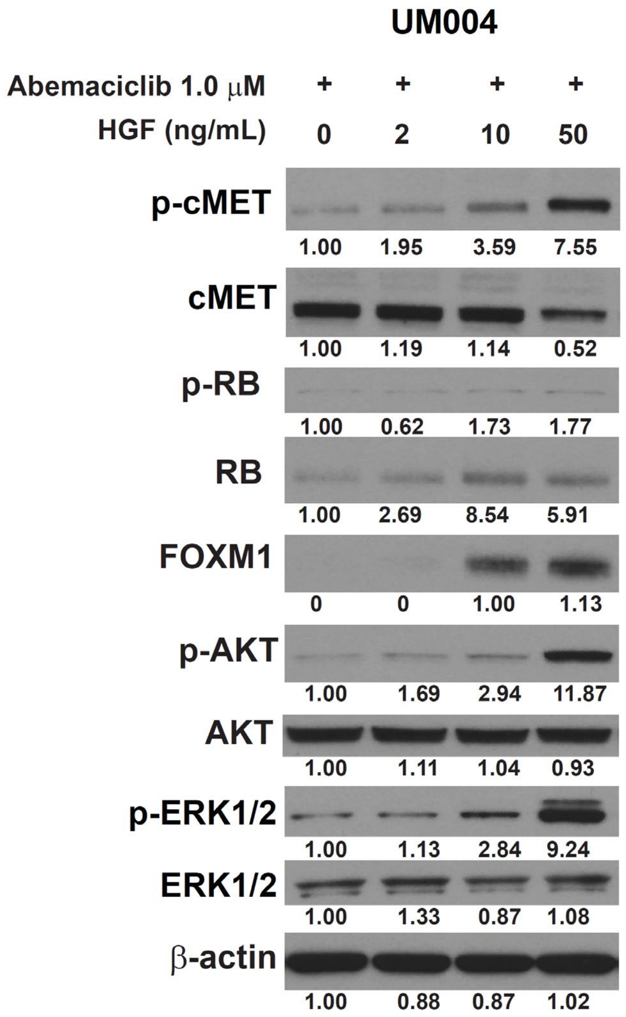

Figure S4. HGF increased FOXM1 in UM004 under abemaciclib treatment. UM004 cells were treated with 1 μM of abemaciclib, in combination with various concentration of HGF as indicated for 72 h. Activation of cMET, RB, FOXM1, AKT, and ERK1/2 was analyzed by Western blotting.

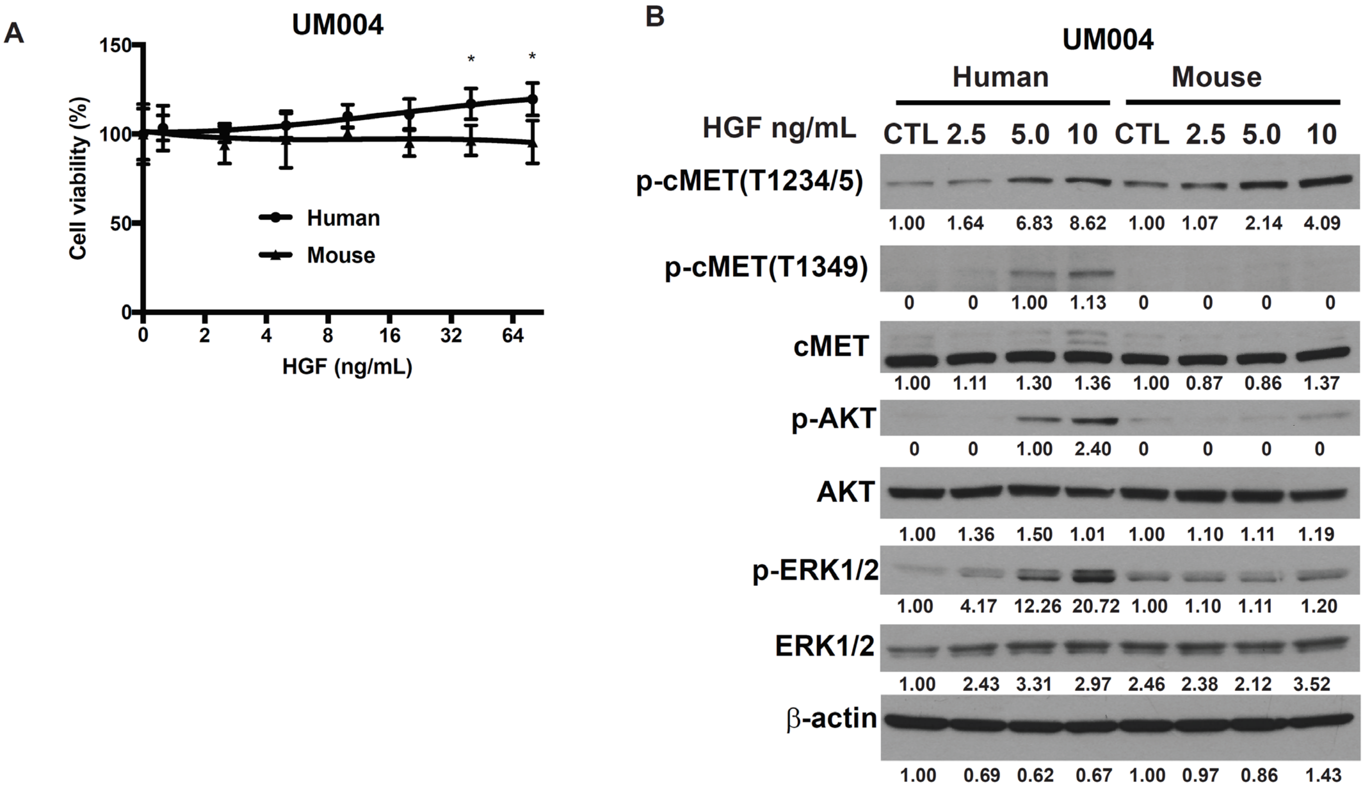

Figure S5. Differential effects of human and mouse HGF on UM004 cells. (**A**) UM004 cells were treated with various concentrations of recombinant human HGF and recombinant mouse HGF as indicated for 96 h. Cell viability was analyzed by the MTS assay. Data represent mean cell viability ± SD. **p* < 0.05; ***p* < 0.01; compared with the group treated with control vehicle based on the two sample *t*-test with unequal variance. **(B**) UM004 cells were treated with different concentrations of recombinant human HGF and recombinant mouse HGF for indicated times. Cells were lysed and Western immunoblotted with the indicated antibodies for phosphorylation of cMET, AKT, and ERK1/2.

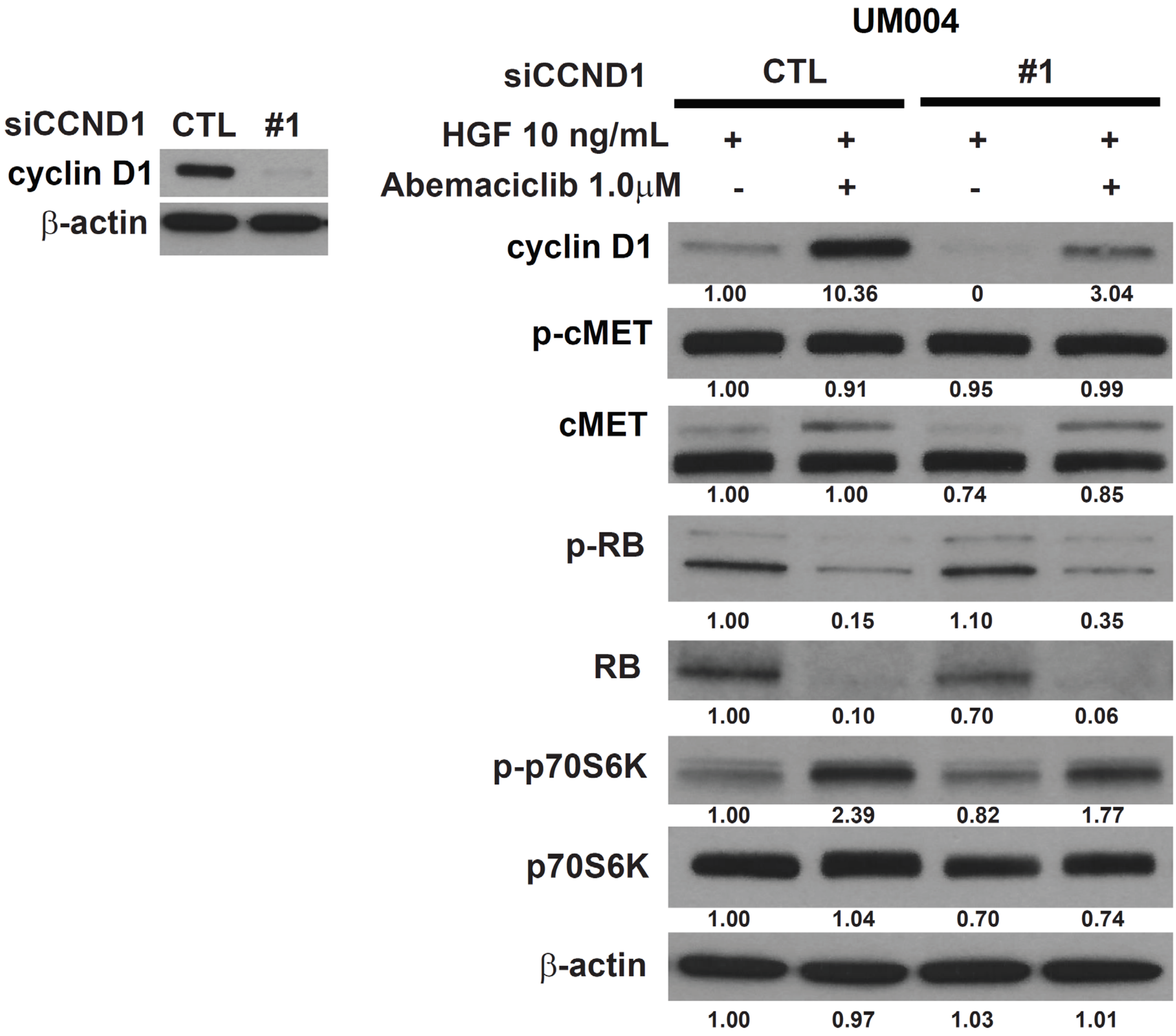

Figure S6. Cyclin D1 has no effect on the activation of p70S6K. UM004 cells were transfected with siRNA targeting cyclin D1 in the presence or absence of 1 μM of abemaciclib and 10 ng/ml of HGF for 48 h. Activation of cMET, RB, and p70S6K was analyzed by Western immunoblotting.

Table S1. Mutational status in the three UM cell lines.

Cell lines	*GNAQ*	*GNA11*	*BAP1*	*SF3B1*	*E1F1AX*
UM001	mut(Q209P)	wt	wt	mut(R625C)	wt
UM002B	wt	mut(Q209L)	wt	wt	wt
UM004	mut(Q209P)	wt	wt	wt	wt

Table S1. Mutational status of *GNAQ*, *GNA11*, *BAP1*, *SFB1*, and *E1F1AX* in the three UM cell lines. UM001, UM002B and UM004 cells harbor *GNAQ* and *GNA11* mutations, as determined by the TruSeq (Illumina, San Diego, CA, USA). No mutations in *BAP1*, *E1F1AX* were identified in any cell lines. UM001 cells harbor *SF3B1* mutations.
